# Clemastine improves hypomyelination in rats with hypoxic–ischemic brain injury by reducing microglia-derived IL-1β via P38 signaling pathway

**DOI:** 10.1186/s12974-019-1662-6

**Published:** 2020-02-15

**Authors:** Di Xie, Xiaoli Ge, Yanli Ma, Jialong Tang, Yang Wang, Yajie Zhu, Chengjin Gao, Shuming Pan

**Affiliations:** Department of Emergency, Xinhua Hospital Affiliated to Shanghai Jiao Tong University School of Medicine, Yangpu District, Shanghai, China

**Keywords:** Clemastine, Interleukin-1β, Oligodendrocyte, Microglia, Hypomyelination

## Abstract

**Background:**

Microglia activation is associated with the development of hypoxic–ischemic brain injury (HIBI). Neuroinflammation suppression might be a suitable therapeutic target in hypoxic oligodendrocyte injury. This study aims to determine whether clemastine can improve hypomyelination by suppressing the activated microglia and promoting the maturation of oligodendrocyte progenitor cells (OPCs) in HIBI.

**Methods:**

A bilateral common carotid artery occlusion (BCCAO) rat model that received continuous intraperitoneal injection (1 mg/kg) for 14 days was employed to elaborate the neuroprotection effects of clemastine. Interleukin-1β (IL-1β), nod-like receptor protein 3 (NLRP3), histamine H1 receptor, and OPC differentiation levels in the corpus callosum were measured. Primary cultured OPCs and co-culture of microglia and OPCs were used to explore the link between microglia activation and hypomyelination. Data were evaluated by one-way ANOVA with Fisher’s protected least significant difference test.

**Results:**

Clemastine treatment could reverse hypomyelination and restrain the upregulation of IL-1β and NLRP3 in the corpus callosum of BCCAO rats. Primary cultured OPCs treated with IL-1β showed failed maturation. However, clemastine could also reverse the OPC maturation arrest by activating the extracellular signal-regulated kinase (ERK) signaling pathway. Co-culture of microglia and OPCs with oxygen glucose deprivation treatment exhibited IL-1β and NLRP3 upregulation. Clemastine could downregulate NLRP3 and IL-1β and reverse hypomyelination by inhibiting the p38 signaling pathway.

**Conclusions:**

Clemastine could restrain microglia activation, improve axonal hypomyelination in BCCAO rats, and thus might be a viable strategy to inhibit hypomyelination in the corpus callosum of patients with HIBI.

## Background

Cardiac arrest can cause severe neurological damage that can lead to a long-term unconscious state, and hypoxic–ischemic brain injury (HIBI) is the main cause of this poor prognosis [1]. White matter damage develops after acute global HIBI; therefore, assessing white matter damage is conducive to determining neurological outcomes [2, 3]. Hypoxic–ischemic white matter damage involves oligodendrocyte injury, which is vulnerable to axonal hypomyelination [4, 5].

Neuroinflammation has been widely recognized as a possible pathological factor in ischemic–hypoxic white matter injury [6] because of its involvement in the activation of microglia and the release of cytotoxic compounds, such as cytokines and reactive oxygen species [7]. Proinflammatory cytokines target oligodendrocyte progenitor cells (OPCs) [8], whereas interleukin-1 beta (IL-1β) impedes OPC recruitment by inhibiting white matter repair and functional recovery [9]. Our previous study showed that excess IL-1β inhibits the maturation of OPCs in the corpus callosum, leading to axonal hypomyelination [10]. Neuroinflammation inhibition may be an interesting therapeutic target for white matter damage. From early damage to tissue repair after ischemia, the exact molecular signaling pathway remains unclear. At present, the role of nod-like receptor protein 3 (NLRP3) inflammasome in neuroinflammation has received considerable attention [11] because the activation of its pathway is involved in microglia-mediated IL-1β production [12]. Therefore, the inhibition of NLRP3 activation in microglia reduces IL-1β release and brain damage [13].

Clemastine is a first-generation histamine H1 receptor (HH1R) antagonist with good safety that features anti-inflammatory effects and improves oligodendrocyte differentiation in hypoxic brain injury [14]. This drug also enhances myelination, rescues behavioral changes, and regulates the imbalance of pro-inflammatory cytokines including IL-1β [15, 16]. Extracellular signal-regulated kinase (ERK) signaling pathway was found to induce OPC maturation [17], and the activation of the former enhances the differentiation of the latter [18]. Whether clemastine improves axonal hypomyelination through the ERK signaling pathway is unclear. p38 is a signaling pathway that regulates IL-1β release [19]. However, the relationship between microglia activation and hypomyelination remains unclear. We hypothesize that clemastine may improve axonal hypomyelination by inhibiting the p38/NLRP3/IL-1β signaling pathway in microglia and activating the ERK signaling in OPCs. This study aims to investigate the molecular mechanism of clemastine in improving axonal hypomyelination after HIBI and to provide a scientific theoretical basis for the individualized targeted clemastine therapy in HIBI.

## Methods

### Animals

Two hundred to 250g male Sprague-Dawley (SD) rats were used in this study. For this study, we used the following experimental groups: (1) several SD rats were sham group. (2) Experimental group performed bilateral common carotid artery ligation (BCCAO). (3) Several SD rats were intraperitoneally injected with clemastine (1mg/kg) after BCCAO. (4) Another group were intraperitoneally injected with the same volume of 0.01 mol/L (M) phosphate-buffer saline (PBS) buffer after BCCAO and were used as age-matched controls. The rats were then allowed to be raised under normoxic conditions for 1, 3, 7, 14, 28 days (d) before being killed. Animal handling and experiments were approved by Institutional Animal Care and Use Committee, Shanghai, China.

### Primary culture of microglial cells

In brief, cerebral hemispheres were separated from 1-day-old postnatal SD rats (obtained from Laboratory Animal Center, Shanghai Jiaotong University, Shanghai China), the meninges and superficial vessels were carefully removed. The cerebral cortex was harvested and digested with 10 ml 0.125% trypsin containing 600 U DNase (Sigma, St. Louis, MO, USA, Cat. No. D4527) for 15 min in 37°C thermostatic water bath. After this, 10 ml of Dulbecco’s modified Eagle’s medium–F12 nutrient mixture (DMEM–F12) (Invitrogen Life Technologies Corporation, Carlsbad, CA, USA; Cat. No. 31330-038) containing 10% fetal bovine serum (FBS) (Invitrogen Life Technologies Corporation; Cat. No. 10099-141) was added. The tissue was then triturated several times with a 5-ml pipette. The un-dissociated tissue clumps were allowed to settle for 1–2 min. Subsequently, the supernatant was collected and passed through a 70-μm cell strainer to remove the remaining small clumps of tissue; the cell suspension was then centrifuged at 1,500 rpm for five minutes. The supernatant was discarded and the cell pellet was resuspended in 10 ml of DMEM–F12 supplemented with 10% FBS. The resuspended cells were seeded into poly-L-lysine-coated 75-cm^2^ flasks at a density of 250,000 cells/ml and cultured at 37°C in humidified 5% CO2/95% air. The medium was changed after 24 h and then replaced every 3–4 days. After 10 days, the bottom of the flask showed a confluency of cells with mixed glia, including mainly microglia/oligodendrocytes. The mixed cells were cultured for 10 days at 37°C and 5% CO_2_ before shaking at 180 revolutions per minute (rpm) and 37°C for 1 h to obtain microglia cells. After incubation in a humidified atmosphere of 95% air and 5% CO2 at 37°C for 24 h, the cells were subjected to different treatments. The microglia cells cultures with above 90% purity were used in this study (details are given in the Additional file 1: Figure S3).

### Treatment of microglial cells culture

Microglial cell cultures were divided into three groups.

- Group I. The purified microglia cells were cultured for 1 day at 5% CO_2_ and 95% air at 37°C, the cells were subjected to different treatments. To study whether clemastine would affect microglial release of inflammatory mediators, microglial cells were treated with (Clemastine group) or without (oxygen glucose deprivation, OGD group) 20ng/ml clemastine for 1 h at 3% oxygen, 5% CO_2_ and 92% nitrogen at 37°C.;control group with equal volume of PBS, Clemastine group were treated with 20ng/ml clemastine, OGD + MCC950 group were treated with MCC950 (0.1μM, Topscience, Cat. No. T6887) for 1 h at 3% oxygen, 5% CO_2_ and 92% nitrogen at 37°C.
- Group II. To study the effect of clemastine on the p38 signaling pathways under hypoxic condition, microglial cells were divided into six groups: Control group, OGD group, OGD + 20ng/ml clemastine group, OGD + 20ng/ml clemastine + 0.1μM MCC950 group, OGD + 20ng/ml clemastine + 0.5μM SB203580 (p38 MAPK inhibitor, Topscience, Cat. No. T1764), 20ng/ml clemastine group. Each group is processed for 1 h. Subsequently, the protein was extracted from the microglial cells and stored at -80°C for Western blotting. In all the experiments, cells were cultured in glucose-free medium except for the control and clemastine group which was cultured in DMEM-F12 containing 10% FBS.
- Group III. To explore whether clemastine would affect microglial release of inflammatory mediators via p38 signaling pathway, microglial cells were divided into six groups: Control group, OGD group, OGD + SB203580 group, OGD + clemastine + SB203580 group, OGD + clemastine + MCC950 group, OGD + clemastine group. Each group is processed for 1 h. Subsequently, the protein was extracted from the microglial cells and stored at -80°C for Western blotting.

### Primary culture of OPCs

The mixed culture is the same as the microglia. The mixed cells were cultured for 10 days at 37°C and 5% CO_2_ before shaking at 180 revolutions per minute (rpm) and 37°C for 1 h to remove microglia cells. The medium was then replaced with fresh DMEM/FBS, and the cultured cells were again shaken at 250 rpm and 37°C for 20 h to harvest OPCs, followed by incubating on a 10-cm Petri dish for 60 min (min) at 37°C to remove contaminating astrocytes and microglia. Purified OPCs were plated on poly-D-lysine (PDL) or laminin-coated coverslips and cultured in oligodendrocyte precursor cell medium (OPCM) (ScienCell Research Laboratories, USA, No.1601) at 5% CO_2_ and 95% air at 37°C. The OPCs cultures with above 90% purity were used in this study (details are given in the Additional file 1: Figure S3).

### Treatment of OPCs culture

OPCs cultures were divided into two groups:

- Group I. To examine the effects of clemastine on the phosphorylation of ERK pathway in the differentiated OPCs. The cells were subdivided into the Control group (0.01M PBS); 30 ng/mL IL-1β group; 30 ng/mL IL-1β + 20 ng/mL clemastine group; 20 ng/mL clemastine group. The OPCs were cultured in oligodendrocyte precursor cell differentiation culture medium (OPCDM) (ScienCell Research Laboratories, USA, No.1631) for 6 h at 5% CO_2_ and 95% air at 37°C.
- Group II. To explore whether clemastine would affect OPCs differentiation via ERK signaling pathway, the OPCs were cultured in OPCDM for 7 days at 5% CO_2_ and 95% air at 37°C. OPCs in group II were divided into Control group (0.01M PBS); 30 ng/mL IL-1β group; 30ng/mL IL-1β + 20ng/mL clemastine + SCH772984 group (a specific inhibitor of ERK pathway, 0.1μM, Topscience, Cat. No. T6066). Each group is processed for 7 days. Subsequently, the protein was extracted from the microglial cells and stored at -80°C for Western blotting.

### Co-culture experiment with microglial cells and OPCs

The purified microglial cells were plated on tissue culture inserts for 6-well plates (0.4μm, Millipore, Cat. No. MCHT06H48) at a density of 1×10^6^ cells. The microglial cells were incubated for 1 h in the presence or absence of clemastine (20 ng/mL), SB203580 (0.5μM) or MCC950 (0.1 μM). In order to minimize OPCs damage due to pretreatment of OGD challenge, we have chosen to treat the target drugs (clemastine, SB203580 and MCC950) before OGD challenge. Each tissue culture insert was placed on the OPCs in 6-well plates. Co-culture system were treated for 1 h at 3% oxygen, 5% CO_2_ and 92% nitrogen at 37°C, then, cultured in OPCDM at 5% CO_2_ and 95% air at 37°C (details are given in the Additional file 1: Figure S2).

### Treatment of co-culture

Co-cultures were divided into two groups:

- Group I. To examine the effects of clemastine on the demyelination after OGD. The co-culture cells were subdivided into the Control group (0.01M PBS); OGD group; OGD + clemastine group; OGD + clemastine+ IL-1 receptor antagonist (30 ng/mL IL-Ra, Peprotech Inc Cat. No. 200-01RA) group.
- Group II. To explore whether clemastine would affect OPCs differentiation via p38 signaling pathway, The co-culture cells were subdivided into Control group, OGD group, OGD + SB203580 group, OGD + clemastine + SB203580 group, OGD + clemastine + MCC950 group, OGD + clemastine group.

### Western blot

Proteins were extracted from the corpus callosum or from primary cell culture using a protein extraction kit (Pierce Biotechnology Inc, IL) according to the manufacturer’s protocol. Protein concentrations were determined by the Bradford method using bovine serum albumin (BSA) (Sigma-Aldrich, St Louis, MO) as a standard. Samples of supernatants containing 50 μg of protein were heated to 95°C for 10 min and were separated by sodium dodecyl sulfate–polyacrylamide gel electrophoresis in 10% gel in a Mini-Protein 3 apparatus (Bio-Rad Laboratories, Hercules, CA). Protein bands were electroblotted onto 0.45 lm polyvinylidene difluoride membranes (BioRad) at 1.5 mA/cm^2^ of membrane for 1 h in Towbin buffer, pH 8.3, to which 20% (volume/volume (v/v)) methanol had been added. After transfer, the membranes were blocked with QuickBlock™ Blocking Buffer (Cat. P0231, Beyotime, China) for 1 h, then incubated with the primary antibodies according to the manufacturer’s recommendations. The primary antibodies used were as follows: phospho p38 (rabbit polyclonal antibody), total p38 (rabbit polyclonal antibody), phospho ERK1/2 (rabbit polyclonal antibody), total ERK1/2 (rabbit polyclonal antibody), IL-1β (rabbit polyclonal IgG 1:1,000), histamine H1 receptor (HH1R) (Rabbit polyclonal IgG 1:1,000), myelin basic protein (MBP) (rabbit polyclonal IgG 1:1,000), 2’,3’-cyclicnucleotide 3’-phosphodiesterase (CNPase) (rabbit polyclonal IgG 1:1,000), platelet-derived growth factor receptor-α (PDGFR-α) (rabbit polyclonal IgG 1:1,000), Olig2 (rabbit polyclonal IgG 1:1,000), Nod-like receptor protein 3 (NLRP3) (rabbit polyclonal IgG 1:1,000), β-actin (rabbit polyclonal IgG 1:1,000) [all from Bioworld Technology, Inc. Cat. No. BS6382, BS3567, BS5016, BS1968, BS3506, BS2733, BS64165, BS90315, BS91039, BS90984, BS90949, AP0731], chondroitin sulfate proteoglycan (NG2) (mouse monoclonal IgG 1:1,000) (Chemicon International; Cat. No. MAB5384). After three washes with TBST, the membranes were incubated with the horseradish peroxidase (HRP)-conjugated secondary antibodies (Cell Signaling Technology; Cat. No. 7074 (anti-rabbit IgG), 7076 (anti-mouse IgG)) for 1 h. The immunoblots were developed using the enhanced chemiluminescence detection system (Pierce Biotechnology Inc, Rockford, IL). Blots were stripped with stripping buffer (Cat. P0025N, Beyotime, China) and hybridized with total kinases or β-actin. The signal intensity of these proteins levels relative to control was measured with Quantity One Software, version 4.4.1 (BioRad Laboratories).

### Double immunofluorescence

The locations of photos are in appendix (Additional file 1: Figure S1). Immunofluorescence intensity is expressed by mean fluorescence intensity via Image J. The sections from control and BCCAO rats (in each group at 1, 3, 7 and 14 days) were divided into four groups. The sections in group I from control and BCCAO rats at 1, 3 and 7 days were then incubated with antibodies directed against anti-IL-1β (rabbit polyclonal IgG 1:500) (Bioworld Technology, Inc. Cat. No. AB1832P) or anti-histamine H1 receptor (rabbit polyclonal IgG 1:200) or NLRP3 (rabbit polyclonal IgG 1:200) and Iba-1 (mouse monoclonal IgG 1:100) (Servicebio, Cat. No. GB12105). The sections in group II from control and BCCAO rats at 14th day were incubated with NG2 (mouse monoclonal IgG 1:100) (Chemicon International; Cat. No. MAB5384) or PDGFR-α (Rabbit polyclonal IgG 1:200) (Bioworld Technology, Inc. Cat. No.BS91039).

The sections in group III from control and BCCAO rats at 14th day were incubated with CNPase (mouse monoclonal IgG 1:1,000) (Chemicon International; Cat. No. NE1020) or MBP (mouse monoclonal IgG 1:1000) (Chemicon International; Cat. No. NE1019). The incubation for all groups was carried out at 4°C overnight. On the following day, the sections were washed and incubated with a secondary antibody: Alexa Fluor 555 goat anti-rabbit IgG (1:100, Life, Cat. No. A21428) or Alexa Fluor 555 goat anti-mouse IgG (1:100, Life, Cat. No. A21424) or FITC goat anti-mouse IgG (Bioworld Technology, Inc. Cat. No. BS50950) at room temperature for 1 h. The ratio of NG2-positive oligodendrocytes was calculated and averaged. The ratio of NG2 and PDGFR-α positive oligodendrocytes in the corpus callosum was calculated by counting four randomly selected microscopic fields in sections obtained from each rat at ×40 objective by a blinded observer.

For cultured microglia cells, the cells were treated with oxygen glucose deprivation (OGD) and OGD+clemastine for 1 h, respectively (for IL-1β or HH1R and NLRP3 detection), and then fixed in 4% paraformaldehyde for 30 min, blocked in 1% BSA for 30 min and incubated with primary antibodies overnight at 4°C. Immunofluorescence labeling was carried out by using primary antibodies directed against anti-IL-1β (Rabbit polyclonal IgG 1:500) (Bioworld Technology, Inc. Cat. No. AB1832P) or anti-histamine H1 receptor (rabbit polyclonal IgG 1:200) or NLRP3 (rabbit polyclonal IgG 1:200) and Iba-1 (mouse monoclonal IgG 1:100) (Servicebio, Cat. No. GB12105) for microglia cultured for 1h, The cells were then incubated with Alexa Fluor 555 goat-anti-rabbit secondary antibody (1:100, Life, Cat. No. A21428) and FITC goat anti-mouse IgG (Bioworld Technology, Inc. Cat. No. BS50950) for 1 h. Finally, the cells were counterstained with DAPI and examined under a fluorescence microscope (Olympus System Microscope Model BX53, Olympus Company Pte, Tokyo, Japan).

For cultured OPCs, the cells were treated with IL-1β or clemastine and IL-1β + clemastine, respectively, and the same volume of PBS as control for 7 days (for NG2 and MBP detection), then fixed in 4% paraformaldehyde for 30 min, blocked in 1% BSA for 30 min and incubated with primary antibodies overnight at 4°C. Immunofluorescence labeling was carried out by using primary antibodies directed against NG2 (mouse monoclonal IgG 1:1,000) (Chemicon International; Cat. No. MAB5384) and MBP (mouse monoclonal IgG 1:1,000) (Chemicon International; Cat. No. NE1019), respectively, for OPCs cultured for 7 days. The cells were then incubated with FITC-conjugated goat-anti-mouse secondary antibody (1:100 Chemicon International, Cat. No. AP130F) or goat anti-mouse IgG Cy3 (1:100, Bioworld Technology, Inc. Cat. No. BS10006) for 1 h. Finally, the cells were counterstained with DAPI and examined under a fluorescence microscope (Olympus System Microscope Model BX53, Olympus Company Pte, Tokyo, Japan). Quantitative analysis of the ratio of NG2, and MBP-positive cells was carried out through counting four randomly selected microscopic fields at ×40 objective by a blinded observer. The percentage of cells with positive expression for the respective antibodies was calculated and averaged. Each experiment was done in triplicate.

### Electron microscopy

The BCCAO rats, BCCAO + clemastine rats at 28 days and their corresponding control rats were perfused with a mixed aldehyde fixative composed of 2% paraformaldehyde and 3% glutaraldehyde in 0.1M phosphate buffer, pH 7.2. After perfusion, the brain was removed and coronal slices (approximately 1 mm thick) were cut. Blocks of corpus callosum were trimmed from these slices. Vibratome sections (Model 3000TM, The Vibratome Company, St. Louis, MO) of 80–100 mm thickness were prepared from these blocks and rinsed overnight in 0.1M phosphate buffer. They were then post-fixed for 2 h in 1% osmium tetraoxide, dehydrated, and embedded in Araldite mixture. Ultrathin sections were cut and viewed in a Philips CM 120 electron microscope (FEI Company, Hillsboro, OR). Four non-overlapping regions of the medial corpus callosum from each animal were photographed at two different magnifications. The diameter of each axon as well as that of axon plus its associated myelin sheath was measured by using Image J software (SummaSketch III Summagraphics, Seattle, WA). The g-ratio was calculated per axon as axon diameter to total axonal fiber diameter [it is equivalent to axon area/(axon+myelin sheath area)] by a researcher blind to control/BCCAO/clemastine injection group and compared among groups with one-way ANOVA (*α* =0.05, two-tailed; *n* =3 per group).

### Statistical analysis

All data were evaluated by the SPSS13.0 statistical software (IBM, Armonk, NY). Different statistical methods were applied according to different types of data. The distribution values were expressed as mean±SD. Four-group univariate-factor measurement data were analyzed by one-way ANOVA if the data were homogeneity of variance; otherwise, they were analyzed by Welch ANOVA. Multiple comparisons were analyzed by the least significant difference (LSD) method if the data were homogeneity of variance; otherwise they were analyzed by Dunnett’s T3 method. The criterion for statistical significance was set at *P* < 0.05.

## Results

### Determining IL-1β, NLRP3, and HH1R protein expression levels in the corpus callosum by double labeling and Western blot analysis

**Fig. 1:**
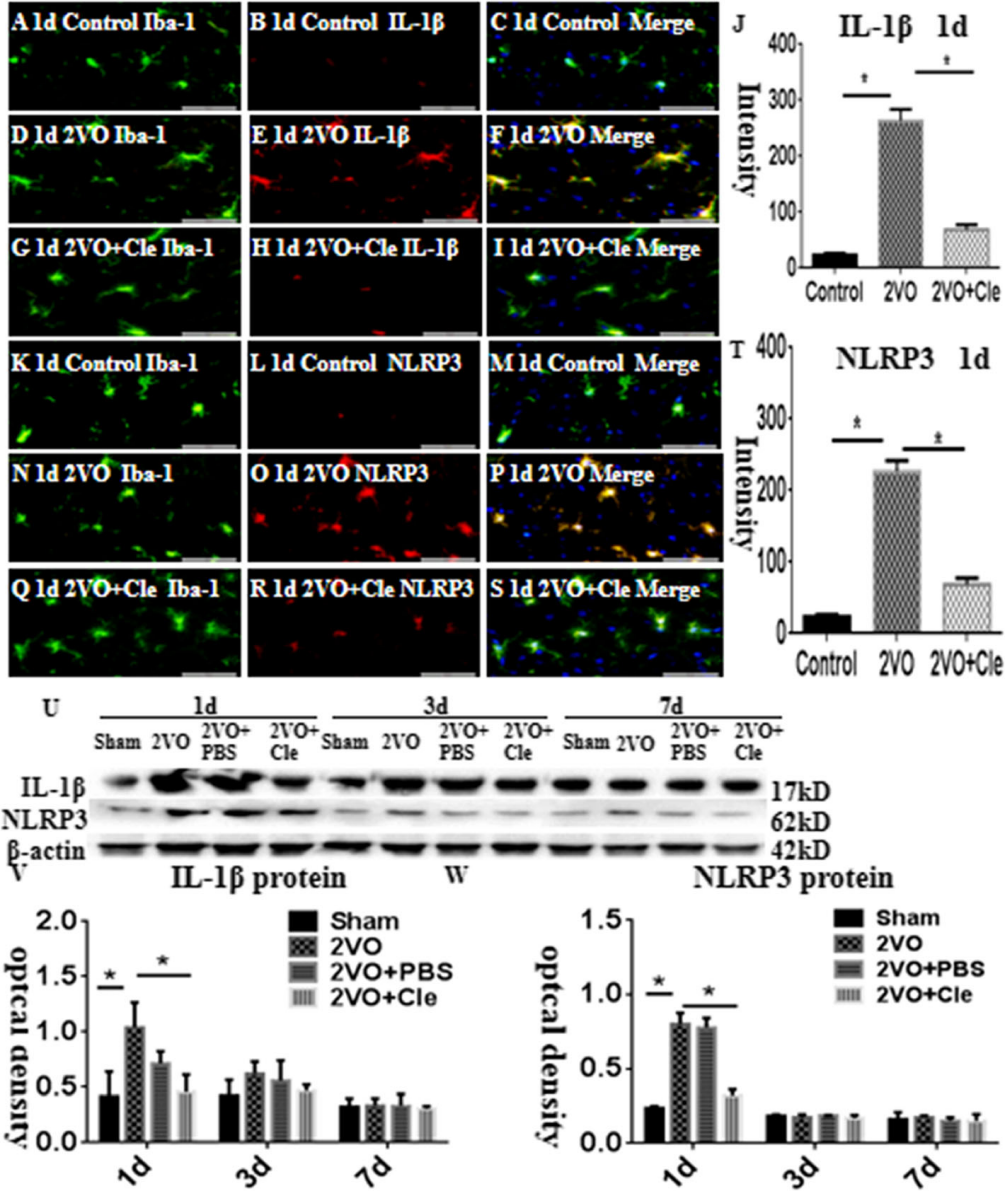
**a**–**w** show interleukin-1beta (IL-1β) and nod-like receptor protein 3 (NLRP3) protein expression levels in the corpus callosum of rats at 1, 3, and 7 days after the bilateral common carotid artery occlusion (BCCAO) and clemastine injection after BCCAO when compared with their corresponding controls. Double immunofluorescence staining showing the distribution of Iba-1-labeled (**a**, **d**, **g**, **k**, **n**, and **q**, green) and IL-1β (**b**, **e**, and **h**, red) and NLRP3 (l, o, and r, red) immunoreactive amoeboid microglial cells (AMCs) in the corpus callosum of rats at 1 day after BCCAO and clemastine injection after BCCAO and their corresponding controls. The colocalized expression of Iba-1 and IL-1β or NLRP3 in AMCs can be seen in **c**, **f**, **l**, or **m**, **p**, **s**. Bar graphs (**j** and **t**) depict remarkable increases in the immunofluorescence intensity of IL-1β and NLRP3 expression levels, following BCCAO challenge when compared with matched controls; clemastine reversed these changes. Panel U shows IL-1β (17 kDa), NLRP3 (62 kDa), and β-actin (42 kDa) immunoreactive bands. Panels V and W show bar graphs depicting significant increases in the optical density of IL-1β and NLRP3 after BCCAO when compared with their corresponding controls. Meanwhile, clemastine could remarkably decrease the expression of IL-1β and NLRP3 at 1day. *N* = 3. *P < 0.05. Scale bars: A–S 50 μm

**Fig. 2:**
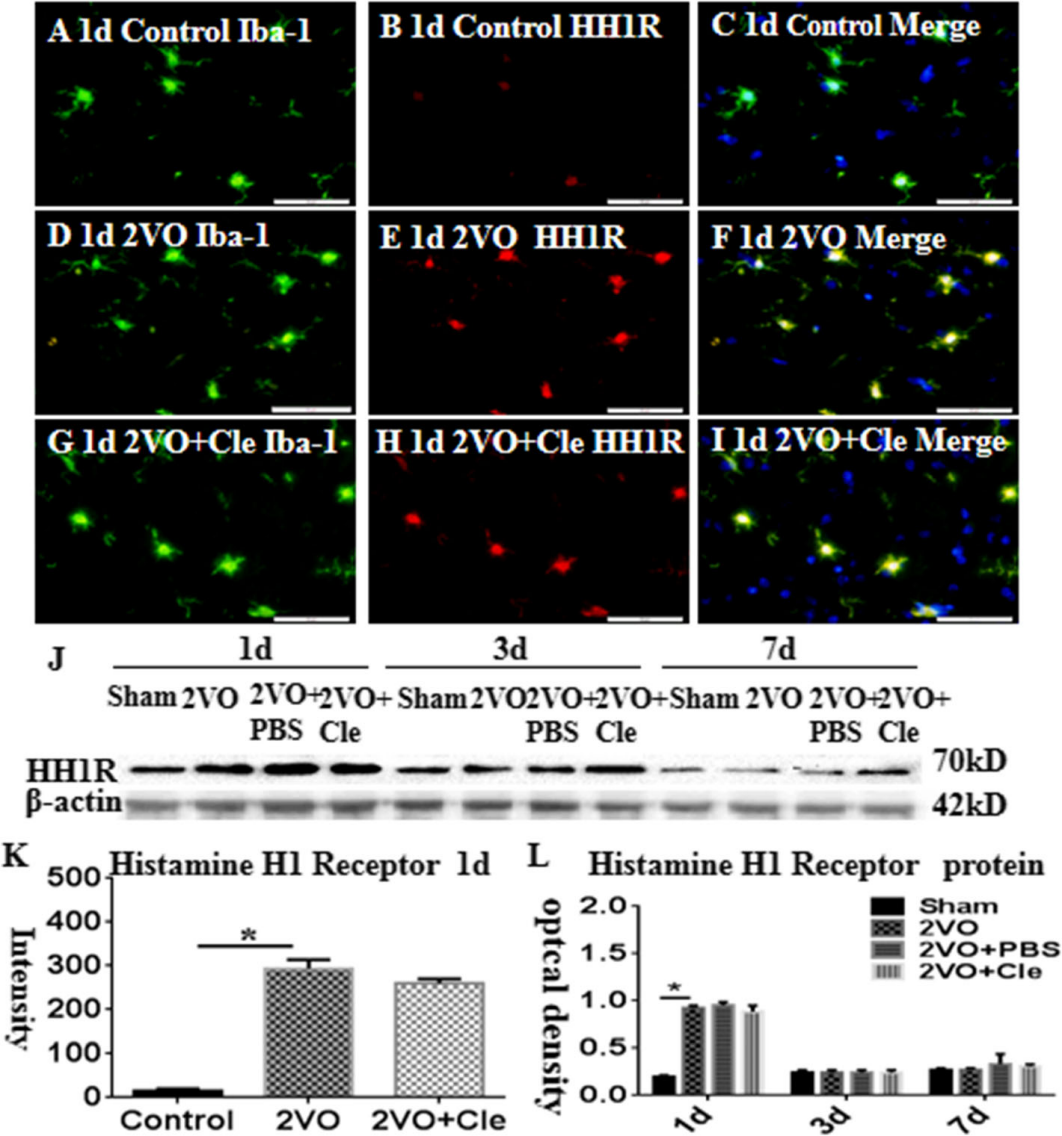
Histamine H1 receptor (HH1R) protein expression in the corpus callosum of rats at 1, 3, and 7 days after the bilateral common carotid artery occlusion (BCCAO) and clemastine injection after BCCAO when compared with their corresponding controls. Double immunofluorescence staining showing the distribution of Iba-1-labeled (**a**, **d**, and **g**, green) and HH1R (B, E, and H, red) immunoreactive amoeboid microglial cells (AMCs) in the corpus callosum of rats at 1 day after BCCAO and clemastine injection after BCCAO and their corresponding controls. The colocalized expression of Iba-1 and HH1R in AMCs can be seen in **c**, **f**, and **i**. Panel **j** shows HH1R (70 kDa) and β-actin (42 kDa) immunoreactive bands. Panels K and L show bar graphs depicting significant increases in the immunofluorescence intensity and optical density of HH1R following the BCCAO when compared with their corresponding controls; clemastine showed no effect on the expression of HH1R. *N* = 3. *P < 0.05. Scale bars: A–I 50 μm.

In the corpus callosum of the control rats, the interleukin-1beta (IL-1β) and NLRP3 expression levels were specifically detected in sporadic cells and confirmed to be microglial cells by double labeling with Iba-1 staining (Fig. 1a–i and k–s). At 1 day following bilateral common carotid artery occlusion (BCCAO), the IL-1β and NLRP3 immunoreactivities were induced in numerous microglial cells (Fig. 1d–f and n–p) when compared with that of the controls (Fig. 1a–c and k–m). However, clemastine reversed these changes (Fig. 1g–i and q–s). The excessive expression levels of IL-1β and NLRP3 decreased after treatment with clemastine (Fig. 1j and t, respectively). The expression of HH1R was localized in the microglial cells as confirmed by double immunofluorescence showing colocalization of Iba-1 staining (Fig. 2a–i). At 1 day following BCCAO, the HH1R immunoreactivity was markedly enhanced in the soma of microglial cells (Fig. 2d–f) compared with that of the control (Fig. 2a–c). However, clemastine caused no effect on its expression (Fig. 2g–i and k). The immunoreactive bands of IL-1β and NLRP3 protein levels, which appeared at approximately 17 and 62 kDa, respectively (Fig. 1u), increased significantly (*P < 0.05) in optical density at 1, 3, and 7 days after BCCAO compared with that of the controls. However, clemastine could reverse this phenomenon (Fig. 1v and w). The immunoreactive bands of HH1R protein levels, which appeared at approximately 70 kDa (Fig. 2k), increased significantly (*P < 0.05) in optical density at 1 day after BCCAO compared with that of the controls; clemastine showed no effect on the expression of HH1R (Fig. 2l).

### Frequency of OPCs in the corpus callosum at 1, 7, and 14 days

**Fig. 3:**
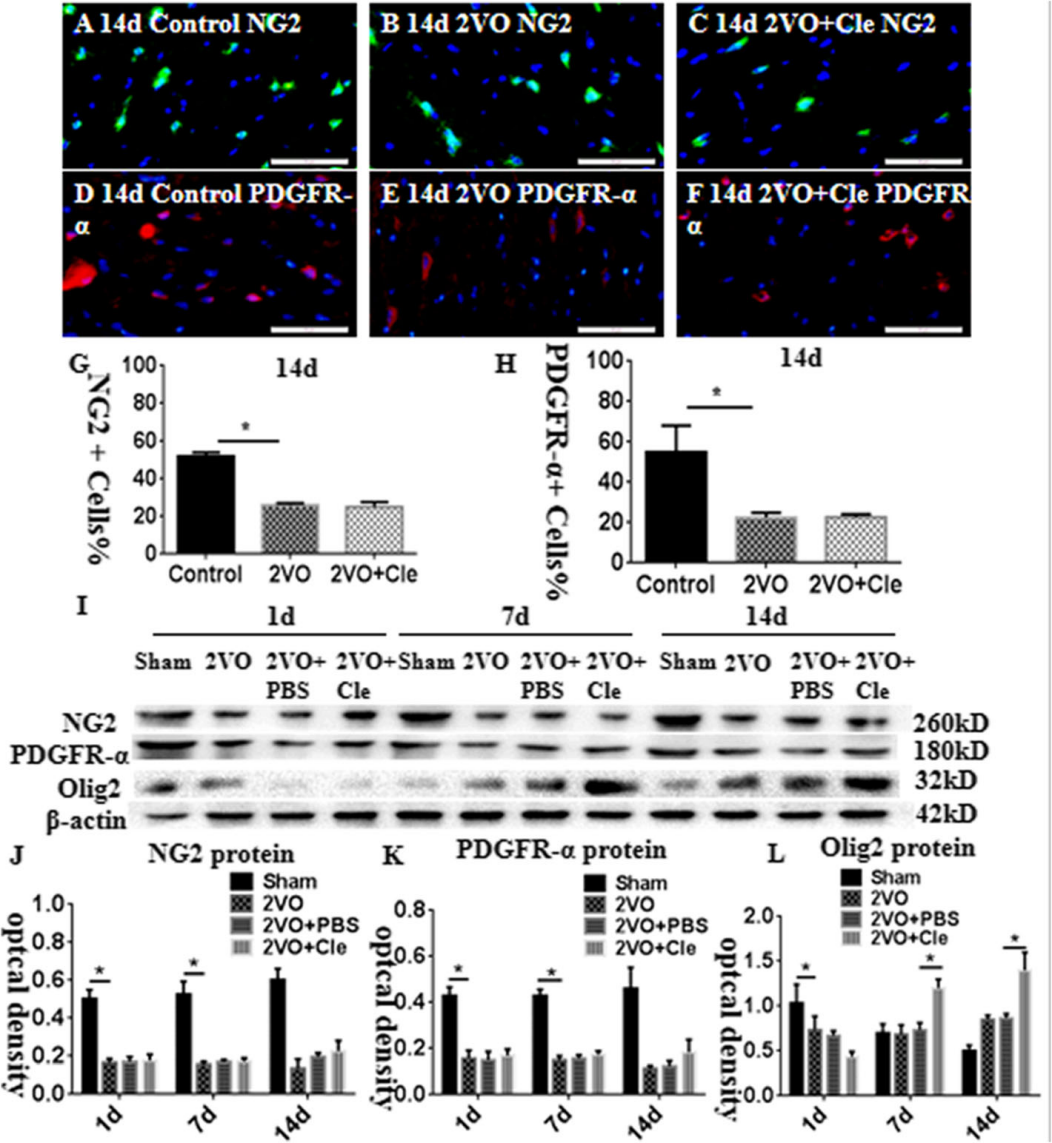
Immunofluorescence staining showing platelet-derived growth factor receptor (PDGFR)-α and neural/glial antigen 2 (NG2) immunoreactive oligodendrocytes (PDGFR-α, red; NG2, green) in the corpus callosum of rats at 14 days after the bilateral common carotid artery occlusion (BCCAO; B and E) and clemastine injection after BCCAO (**c** and **f**) when compared with their corresponding controls (**a** and **d**). Bar graph in G and H shows a remarkable decrease in the ratios of PDGFR-α and NG2-positive oligodendrocytes/mm2 in the corpus callosum at 14 days after the BCCAO when compared with their corresponding controls; clemastine showed no effect on the ratios of NG2 and PDGFR-α. Panel I shows NG2 (260 kDa), PDGFR-α (180 kDa), oligodendrocyte transcription factor 2 (Olig2; 32 kDa), and β-actin (42 kDa) immunoreactive bands. Panels J and K show bar graphs depicting significant decreases in the optical density of NG2 and PDGFR-α at 1, 7, and 14 days following the BCCAO when compared with their corresponding controls; clemastine showed no effect on the expression of NG2 and PDGFR-α. Panel L shows a bar graph depicting a significant decrease in the optical density of Olig2 at 1 day and increase at 7 and 14 days following the BCCAO when compared with their corresponding controls; Olig2 protein increases significantly at 7 and 14 days after treatment with clemastine compared with that of BCCAO. *N* = 3. *P < 0.05. Scale bars: A–F 50 μm.

To observe the differentiation and maturation of OPCs, we used OPC markers, such as platelet-derived growth factor receptor (PDGFR)-α and neural/glial antigen 2 (NG2), to detect the ratio of OPCs in the corpus callosum. The ratio of NG2-positive OPCs remarkably decreased in the corpus callosum at 14 days after BCCAO in comparison with that of the corresponding controls; however, clemastine showed no effect on the expression of NG2 (Fig. 3a–c and g; *P < 0.05) the same with the PDGFR-α (Fig. 3d–f and h; *P < 0.05). The immunoreactive bands of NG2, PDGFR-α, and oligodendrocyte transcription factor 2 (Olig2) proteins, which appeared at approximately 260, 180, and 32 kDa, respectively (Fig. 3i), decreased significantly at 1, 7, and 14 days after BCCAO compared with that of the controls. Clemastine caused no upregulation in the expression levels of NG2 and PDGFR-α (Fig. 3j and k). Olig2 protein decreased significantly in optical density on day 1 and increased at 7 and 14 days after BCCAO compared with that of the controls. However, Olig2 protein increased significantly at 7 and 14 days after treatment with clemastine compared with that of BCCAO (Fig. 3l; *P < 0.05).

### Myelin basic protein (MBP) and 2′,3′-cyclic nucleotide-3′-phosphodiesterase (CNPase) protein expression levels in the corpus callosum

**Fig. 4:**
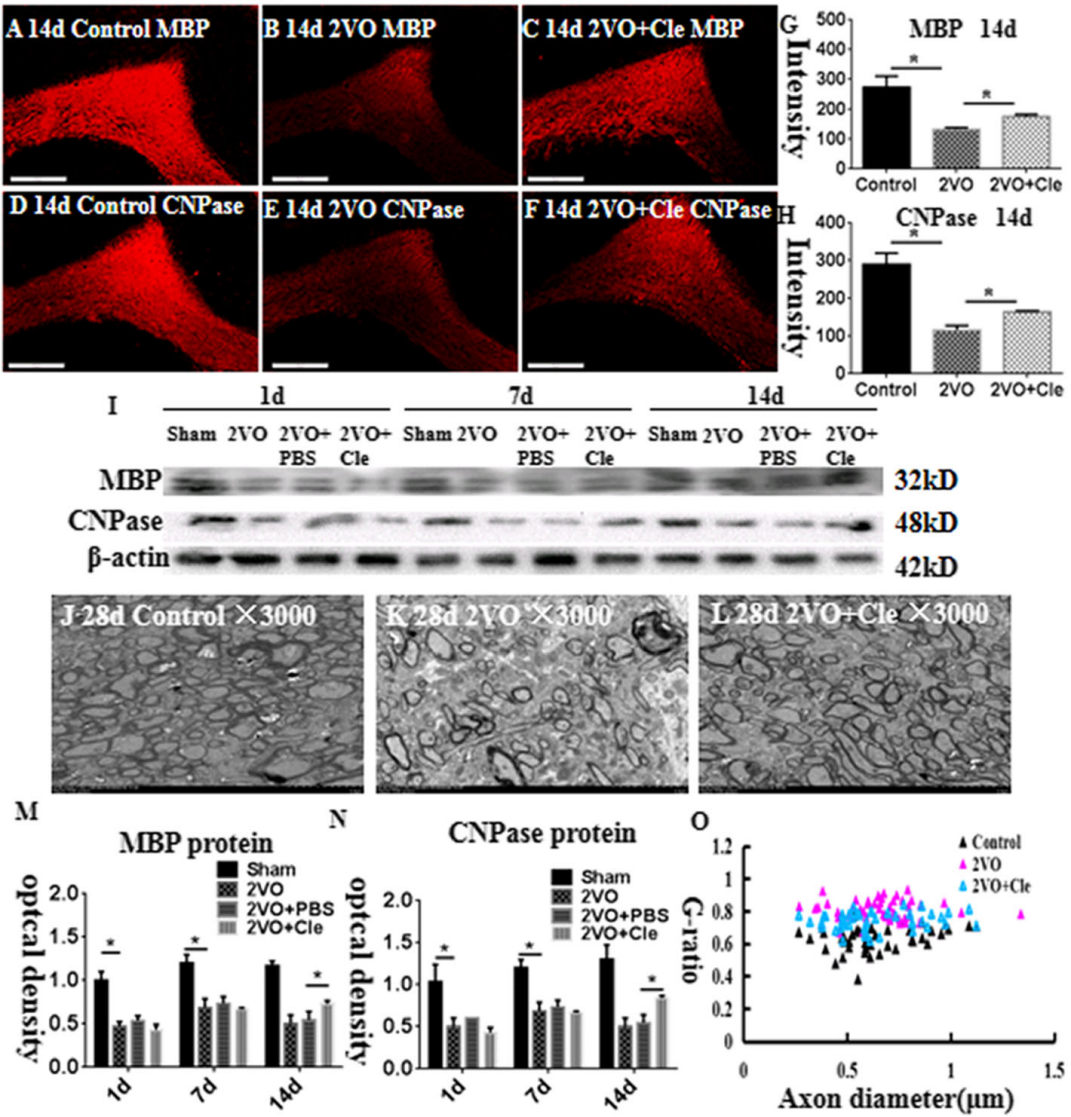
Hypomyelination in the corpus callosum of rats at 14 and 28 days after the bilateral common carotid artery occlusion (BCCAO; **a**–**h**). Immunofluorescence staining showing myelin basic protein (MBP) and 2′,3′-cyclic nucleotide-3′-phosphodiesterase (CNPase) expression in the corpus callosum of rats at 14 days after the BCCAO (**b** and **e**) and clemastine injection after BCCAO (**c** and **f**) and their corresponding controls (**a** and **d**). Bar graph in **g** and **h** shows a significant decrease in the immunofluorescence intensity of MBP and CNPase in the corpus callosum at 14 days after the BCCAO when compared with their corresponding controls; clemastine reversed these changes. Panel I shows MBP (32 kDa), CNPase (48 kDa), and β-actin (42 kDa) immunoreactive bands. Panels **m** and **n** show bar graphs depicting significant decreases in the optical density of MBP and CNPase at 1, 7, and 14 days following the BCCAO when compared with their corresponding controls; clemastine reversed these changes at 14 days (*P < 0.05). **j**–**l** Electron micrographs showing hypomyelination and aberrant ensheathment of axons in the corpus callosum at 28 days after the BCCAO. The number of myelinated axons in the corpus callosum of 28-day rats in BCCAO group (**k**) decreases remarkably when compared with their corresponding controls (**j**), treated with clemastine which could reverse the hypomyelination after the BCCAO (**l**). O Scatter plots of g-ratios against axon diameters in the corpus callosum at 28 days are shown. G-ratios increased after BCCAO when compared with their corresponding controls; clemastine reversed the changes, indicating that clemastine could promote myelin formation in the myelinated fibers. *N* = 3. Scale bars: **a**–**f**, 200 μm; **j**-**l**, 10 μm

MBP and CNPase are useful and specific markers for mature myelin sheath in the central nervous system (CNS). Immunostaining showed that MBP and CNPase protein expression levels decreased in the corpus callosum at 14 days after BCCAO compared with their age-matched littermates (Fig. 4a–c and d–f); however, clemastine could reverse this phenomenon (Fig. 4g and h). The immunoreactive bands of MBP and CNPase protein levels decreased significantly in optical density in the corpus callosum at 1, 7, and 14 days after BCCAO (Fig. 4i). However, clemastine could reverse this phenomenon at 14 days (Fig. 4m and n). These findings indicated that BCCAO can cause hypomyelination, whereas clemastine can alleviate hypomyelination in CNS.

### Ultrastructural study

From the electron microscopy (EM) observation, the packing density of myelinated axons remarkably reduced as manifested by the loosely organized fibers in the corpus callosum at 28 days after BCCAO (Fig. 4k) compared with that of the corresponding controls (Fig. 4j). Remarkably, the myelin of axons was noticeably thin at 28 days after BCCAO (Fig. 4k) in comparison with that of the corresponding controls (Fig. 4j); however, clemastine alleviated this condition (Fig. 4l). The g-ratios were measured by determining the axon diameter/fiber diameter of myelinated axons to analyze the structural deficits quantitatively. The averaged g-ratio in 28-day control rats reached 0.62. The value significantly increased to 0.78 in rats after BCCAO. After the intervention by clemastine, the averaged g-ratio totaled 0.7, indicating that clemastine reversed the reduced myelin thickness in BCCAO rats (Fig. 4j–l and o). Therefore, the EM results demonstrated the few myelinated fibers and thin myelin sheaths in the corpus callosum at 28 days after BCCAO. On the contrary, clemastine could promote myelin formation in the myelinated fibers.

### Administration of clemastine promoted the maturation of primary OPCs in vitro

**Fig. 5:**
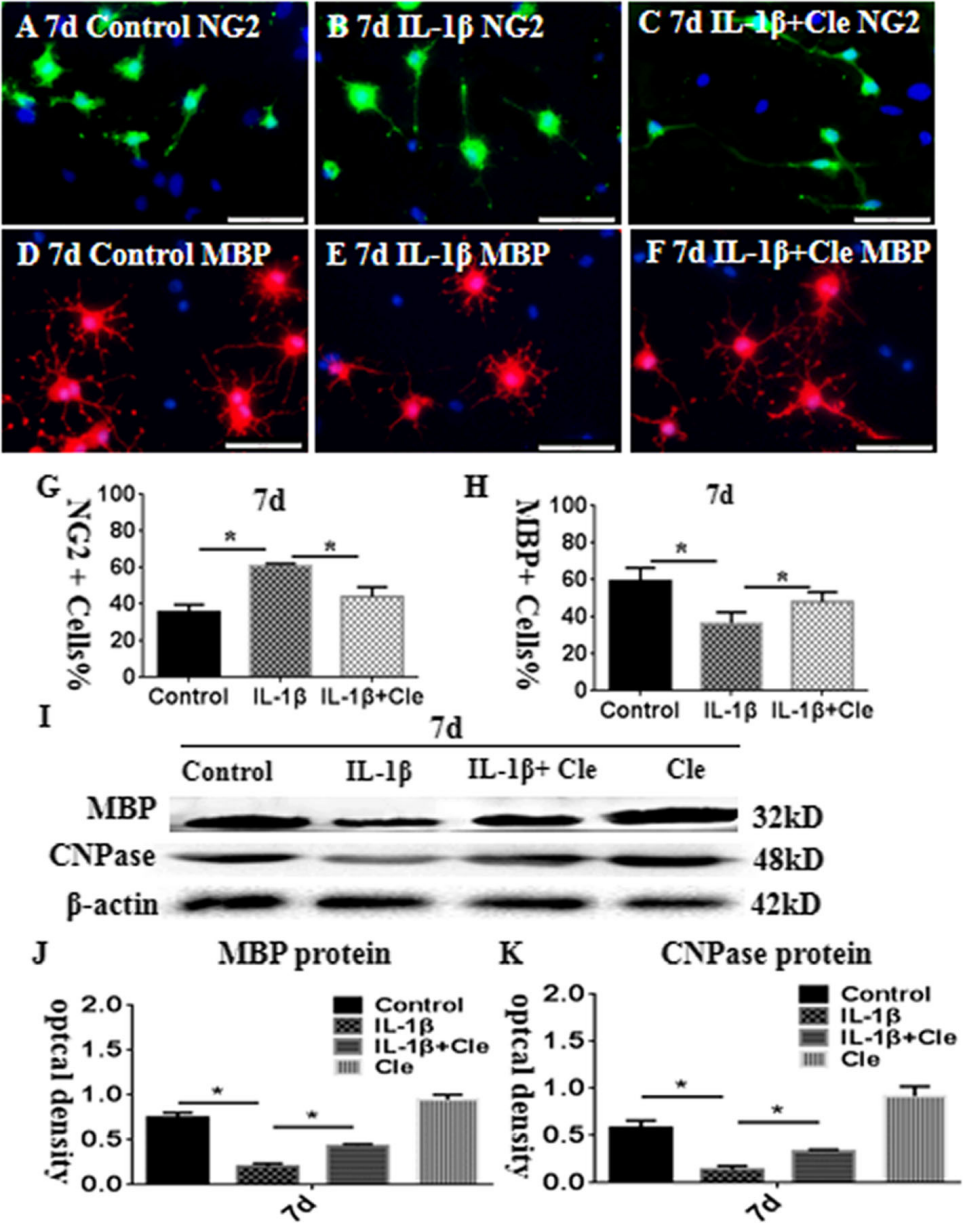
Clemastine promotes differentiation of oligodendrocyte progenitor cells (OPCs) insulted by IL-1β in vitro. Immunofluorescence images of cultured OPCs show the ratios of NG2 (**a**–**c**, green) and MBP (**d**–**f**, red) at 7 days after IL-1β administration (**b**, **e**) or IL-1β + clemastine treatment (**c**, **f**) when compared with their corresponding controls (**a**, **d**). Bar graph in **g** and **h** shows a remarkable increase in the ratio of NG2-positive oligodendrocytes and decrease in the ratio of MBP-positive oligodendrocytes at 7 days after IL-1β administration when compared with their corresponding controls; clemastine reversed these changes. Panel **i** shows MBP (32 kDa), CNPase (48 kDa), and β-actin (42 kDa) immunoreactive bands. Bar graphs in **j** and **k** show significant decreases in the optical density of MBP and CNPase at 7 days after IL-1β administration when compared with their corresponding controls. The clemastine may attenuate the increment. *N* = 3. *P < 0.05. Scale bars: **a**–**f** 50 μm

Immunofluorescence and Western blot were employed to explore the effect of clemastine and measure the maturation of the same number of primary OPCs in the differentiation medium for 7 days after administration with IL-1β, IL-1β+clemastine, and an equal volume of phosphate-buffered saline (PBS) as control. The percentage of NG2-positive OPCs at 7 days following the administration of IL-1β was considerably higher than that in the corresponding controls (Fig. 5a and b). However, clemastine could revert the inhibition of IL-1β on the maturation of primary OPCs (Fig. 5c and g). By contrast, the percentage of MBP-positive oligodendrocytes at 7 days following the administration of IL-1β was considerably lower than that in the matching controls (Fig. 5d and e). However, clemastine could remarkably increase the percentage of mature oligodendrocytes (Fig. 5f and h). The protein expression levels of MBP and CNPase considerably decreased at 7 days after the administration of IL-1β compared with that of the controls (Fig. 5i–k). However, the administration of clemastine could revert the expression of the abovementioned proteins (Fig. 5i–k). Thus, in vitro data showed that clemastine could facilitate the maturation of primary OPCs.

### Clemastine affected the expression of CNPase, MBP, and Olig2 via the activation of ERK phosphorylation in OPCs

**Fig. 6:**
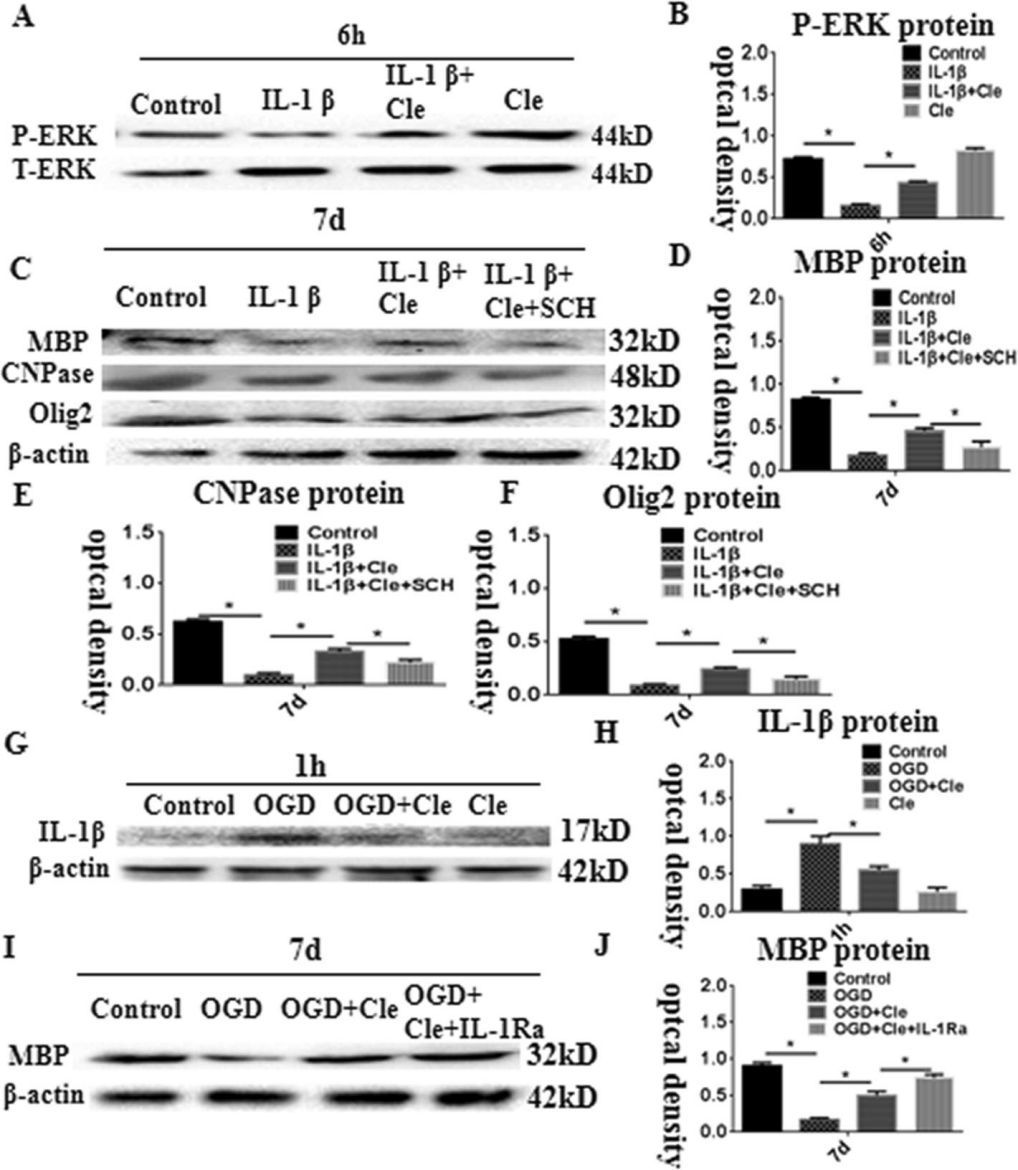
Western blot analysis showing clemastine-upregulated MBP, CNPase, and Olig2 expression via activation of extracellular regulated protein kinases (ERK) pathway in OPCs. Panel **a** showing ERK phosphorylation and total ERK immunoreactive bands. Panel **b** is a bar graph showing significant decreases in the optical density of ERK phosphorylation following treatment with IL-1β; clemastine reversed these changes. Panel **c** shows immunoreactive bands, which indicate that expression of MBP, CNPase, and Olig2 was inhibited in the OPCs after IL-1β administration for 7 days. The clemastine may attenuate the increment. SCH772984 which is an ERK pathway inhibitor could reverse the expression of MBP, CNPase, and Olig2 induced by IL-1β + clemastine treatment. Panels **d**–**f** are bar graphs showing remarkable suppression of MBP, CNPase, and Olig2 by SCH772984. Panel G shows immunoreactive bands, which indicate that expression of IL-1β was increased in the AMCs after oxyglucose deprivation (OGD) administration for 1 h; clemastine could remarkably reduce the expression of IL-1β (**h**). Panel **i** shows immunoreactive bands, which indicate that expression of MBP was decreased in the co-culture of microglia with OPCs at 7 days after oxyglucose deprivation (OGD) administration for 1 h; clemastine could upregulate the expression levels of MBP after OGD. When an IL-receptor antagonist was added, the expression of MBP increased compared with that in the clemastine group after OGD (J). *N* = 3. *P < 0.05

We hypothesized that clemastine promotes the maturation of OPCs through the promotion of ERK phosphorylation. The protein expression levels of CNPase, MBP, and Olig2 were measured by Western blot of OPCs treated with IL-1β + clemastine + SCH772984 (a specific inhibitor of the ERK pathway) at 7 days to ascertain this assumption. The phosphorylation of ERK protein level showed significant downregulation in optical density at 6 h after the administration of IL-1β compared with that of the controls. Meanwhile, clemastine could considerably improve the expression of phosphorylation of ERK (Fig. 6a and b). The protein expression levels of MBP, CNPase, and Olig2 were considerably downregulated at 7 days after treatment with IL-1β compared with that of the controls (Fig. 6c). On the contrary, clemastine could upregulate the expression levels of MBP, CNPase, and Olig2 proteins induced by IL-1β administration by the activation of ERK phosphorylation. The effect of clemastine reduced after adding SCH772984 (Fig. 6c–f). Thus, the in vitro data substantiated that clemastine could promote the maturation of primary OPCs through activation of ERK phosphorylation.

### Clemastine improves axonal hypomyelination by inhibiting microglial activation in vitro

**Fig. 7:**
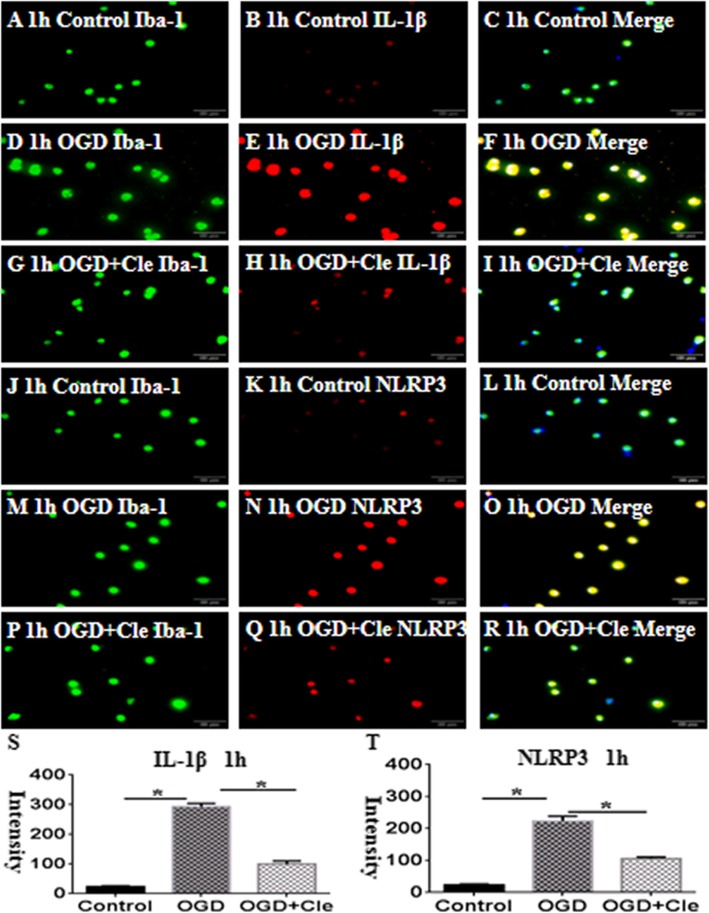
Clemastine inhibits expression of IL-1β and NLRP3 protein of microglial cells insulted by oxygen glucose deprivation (OGD) in vitro. Double immunofluorescence staining showing the distribution of Iba-1-labeled (**a**, **d**, **g**, **j**, **m**, and **p**, green) and IL-1β (**b**, **e**, and **h**, red) and NLRP3 (**k**, **n**, and **q**, red) immunoreactive AMCs in vitro at 1 h after OGD and OGD+clemastine and their corresponding controls. The colocalized expression of Iba-1 and IL-1β or NLRP3 in AMCs can be seen in **c**, **f**, **i**, or **i**, **o**, **r**, respectively. Bar graphs (**s** and **t**) depicting significant increase in the immunofluorescence intensity of IL-1β and NLRP3 expression, respectively, following OGD challenge when compared with matched controls, clemastine reversed these changes. *N*=3. *P < 0.05. Scale bars: **a**–**r** 100μm

**Fig. 8:**
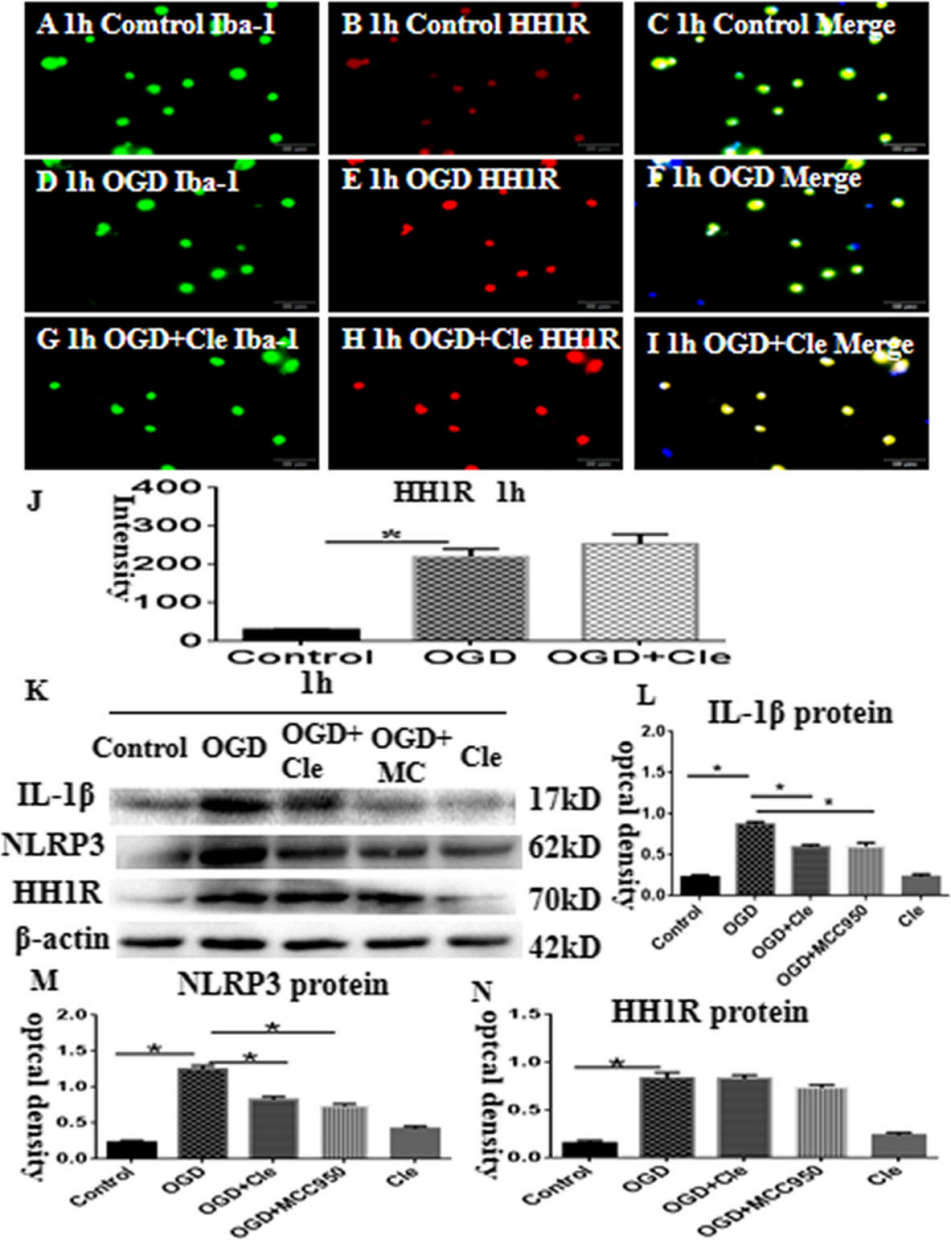
Clemastine inhibits expression of IL-1β and NLRP3 and exerts no effect on the expression of HH1R of microglial cells in vitro. Double immunofluorescence staining showing the distribution of Iba-1-labeled (**a**, **d** and **g**, green) and HH1R (**b**, **e** and **h**, red) immunoreactive AMCs in vitro at 1 h after OGD and OGD + clemastine and their corresponding controls. The colocalized expression of Iba-1 and HH1R in AMCs can be seen in **c**, **f**, and **i**. Panels **j** and **n** show bar graphs depicting significant increases in the immunofluorescence intensity and optical density of HH1R following the OGD when compared with their corresponding controls, clemastine and MCC950 showed no effect on the expression of HH1R. Panel **k** showing IL-1β (17 kDa), NLRP3 (62 kDa), HH1R (70 kDa), and β-actin (42 kDa) immunoreactive bands. Panels **k** and **l** are bar graphs which indicate that expression of IL-1β and NLRP3 were improved in the AMCs after OGD administration for 1h. The clemastine and MCC950 may attenuate the increment. *N*=3. *P < 0.05. Scale bars: **a**–**i** 50μm

Co-culture of microglia with OPCs was established to explore the effect of clemastine on the activation of microglial cells and axonal hypomyelination in vitro. The immunoreactive bands of IL-1β protein levels appeared at approximately 17 kDa and increased significantly in optical density at 1 h after oxyglucose deprivation (OGD) compared with that of the controls. However, clemastine could remarkably reduce the expression of IL-1β (Fig. 6g and h) and upregulate the expression levels of MBP after OGD. When an IL-receptor antagonist was added, the expression of MBP increased compared with that in the clemastine group after OGD (Fig. 6i and j). Double immunofluorescence showed higher expression levels of IL-1β and NLPR3 characterized at 1 h after OGD (Fig. 7d–f and m–o, respectively) in comparison with that of the controls (Fig. 7a–c and j–l). On the contrary, clemastine could remarkably reduce the expression levels of IL-1β and NLRP3 (Fig. 7g–i and p–t). The protein expression levels of IL-1β and NLRP3 considerably increased at 1 h after administration of OGD compared with that of the controls (Fig. 8k–m). The administration of clemastine or MCC950 could revert the expression levels of IL-1β and NLRP3 proteins (Fig. 8k–m). Thus, in vitro data showed that clemastine could inhibit the activation of microglial cells.

### Clemastine caused no effect on the expression of HH1R in microglial cells

Double immunofluorescence was performed to explore the effect of clemastine on the expression of HH1R in microglial cells in vitro. In addition, a higher expression of HH1R was observed at 1 h after OGD (Fig. 8d–f) compared with that of the controls (Fig. 8a–c). Moreover, clemastine did not affect the expression of HH1R (Fig. 8g–j), which was similar to the results of Western blot (Fig. 8k and n). Thus, the in vitro data showed that clemastine does not influence the expression of HH1R in microglial cells.

### Clemastine improves axonal hypomyelination via inhibition of P38 phosphorylation in microglial cells

**Fig. 9:**
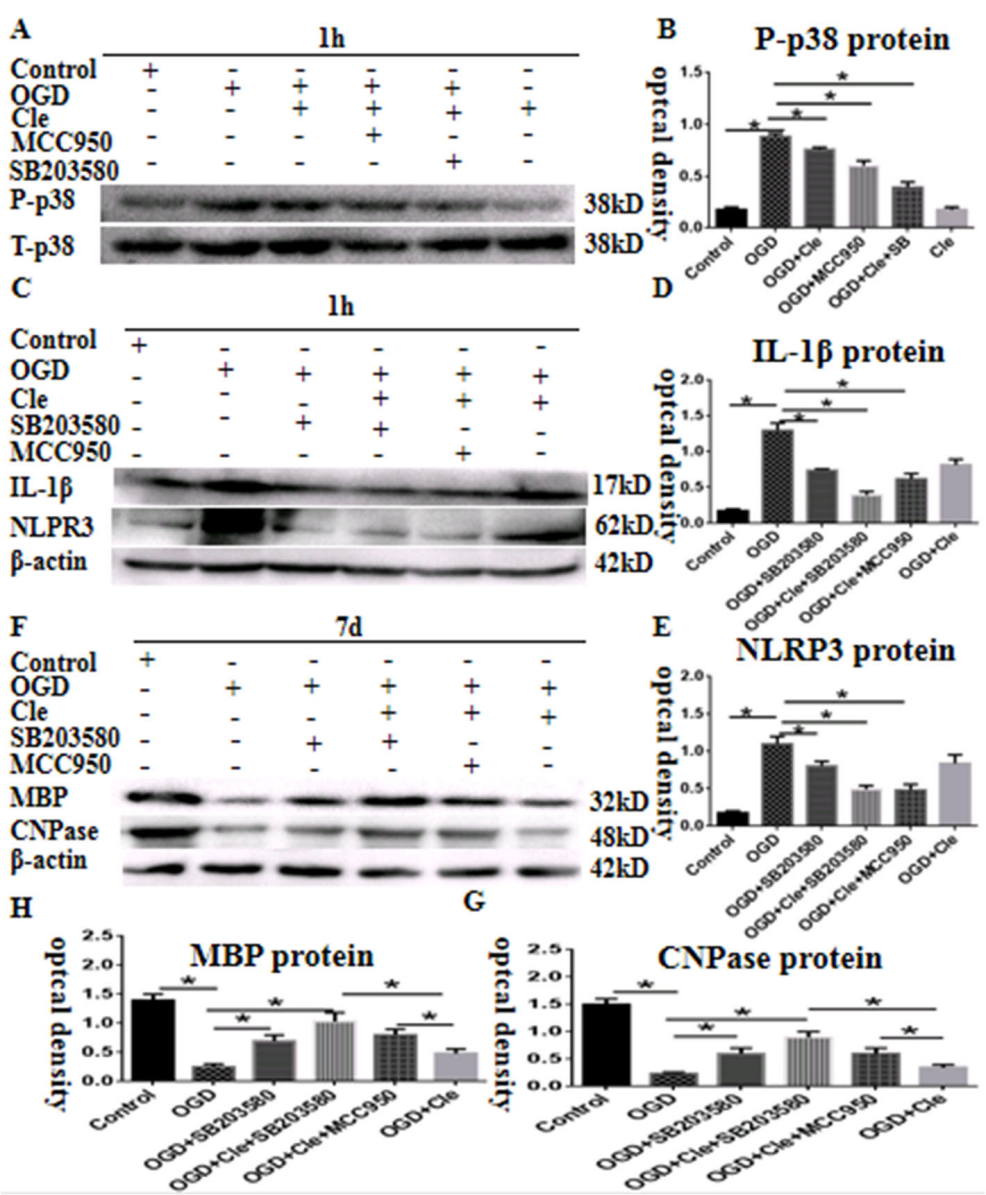
Clemastine improves axonal hypomyelination via inhibition of p38 phosphorylation in microglial cells. Panel **a** showing phosphorylation-p38 (38 kDa) and total-p38 (38 kDa) immunoreactive bands. Panel **b** is bar graph which indicates that expression of p-p38 were improved in the AMCs after OGD administration for 1 h. The clemastine and MCC950 may attenuate the increment. Panel **c** showing IL-1β (17 kDa), NLRP3 (62 kDa), and β-actin (42 kDa) immunoreactive bands. Panels **d** and **e** are bar graphs showing the expression of IL-1β and NLRP3 were significantly up-regulated at 1 h after treatment with OGD compared with the controls. Clemastine could reduce the high expressions of NLRP3 and IL-1β proteins by inhibiting the p-p38. The inhibition was strengthened after adding additional MCC950 or SB203580 (a specific p38 pathway inhibitor). Panel **f** showing MBP (32 kDa), CNPase (48 kDa), and β-actin (42 kDa) immunoreactive bands. Panels **h** and **g** are bar graphs showing the protein expressions of MBP and CNPase were significantly downregulated at 7 days after OGD compared with the controls. Clemastine and SB203580 could upregulate the expressions of MBP and CNPase proteins induced by OGD by inhibiting the p-p38. When additional MCC950 or SB203580 was added, the expression of MBP was upregulated compared with the OGD + clemastine group. *N*=3. *P < 0.05

We hypothesized that clemastine inhibits the activation of microglial cells through the inhibition of p38 phosphorylation. To ascertain this assumption, the protein expression levels of NLRP3 and IL-1β were measured by Western blot in microglial cells at 1 h. The phosphorylation level of p38 protein was significantly upregulated in optical density at 1 h after the administration of OGD compared with that of the controls. However, clemastine and MCC950 could remarkably reduce the expression of phosphorylated p38 (Fig. 9a and b). The protein expression levels of NLRP3 and IL-1β were remarkably upregulated at 1 h after treatment with OGD compared with that of the controls (Fig. 9c). On the contrary, clemastine could reduce the high expression levels of NLRP3 and IL-1β proteins by inhibiting the phosphorylation of p38. The inhibition was strengthened after adding additional MCC950 or SB203580 (a specific p38 pathway inhibitor; Fig. 9c–e). Co-culture of microglia with OPCs was established to examine the link between hypomyelination and the p38 signaling pathway. The protein expression levels of MBP and CNPase were remarkably downregulated at 7 days after OGD compared with that of the controls (Fig. 9f). However, clemastine and SB203580 could upregulate the expression levels of MBP and CNPase proteins induced by OGD by inhibiting the phosphorylation of p38. When additional MCC950 or SB203580 was added, the expression of MBP was upregulated compared with the OGD + clemastine group. Thus, the in vitro data substantiated that clemastine could improve axonal hypomyelination by inhibiting p38 phosphorylation in microglial cells.

## Discussion

Hypoxia may initiate an inflammatory response in the CNS and generate excess amounts of inflammatory mediators that can induce brain injury [20]. Activated microglia elicit neuroinflammation and release inflammatory cytokines such as IL-1β [21]. IL-1β can lead to the dysdifferentiation of OPCs and hypomyelination of axons [22]. In addition, NLRP3 is an important factor in regulating the release of IL-1β [23]. The results showed a remarkable and progressive increase in the protein expression levels of IL-1β and NLRP3 in BCCAO rats up to 1 day. Double immunofluorescence staining has shown that NLRP3 and IL-1β expression levels which are localized in the microglia activated after ischemia–hypoxia and produced excess amounts of IL-1β. HH1R, which exists in the microglia, is associated with neuroinflammation [24]; therefore, blocking the effect of histamine can alleviate neuroinflammation [25]. The present results showed that the expression levels of IL-1β and NLRP3 were decreased by clemastine (H1 receptor antagonist), but the expression of HH1R was unaffected and localized in the microglia. The OPCs include NG2 and PDGFR-α cells, which differentiate progressively in a multiprocess morphology and sequentially bind to anti-CNPase and anti-MBP antibodies [26–28]. Through differentiation and maturation, OPCs migrate to myelinated target axons to form myelin. The maturation of OPCs plays a crucial role in the myelination of targeting axons. Several transcription factors are involved in the maturation of OPCs. Specifically, Olig2 promotes the differentiation of OPCs [29, 30]. The present results showed that the ratio of NG2-positive OPCs remarkably decreased in the corpus callosum at 14 days after ischemia–hypoxia, indicating that clemastine caused no effect on the OPC expression. In addition, the level of Olig2, which promoted the maturation of OPCs, increased at 7 and 14 days, indicating that clemastine may also promote the maturation of OPCs. The present data suggested that the expression levels of MBP and CNPase were downregulated in the corpus callosum, and the proportion of myelinated axons and thin myelin sheath decreased at 14 days after ischemia–hypoxia. These results indicated that the myelin sheath formation was disrupted. Clemastine is an H1 receptor antagonist and promotes the differentiation of OPCs [31–33]. We observed that clemastine can inhibit the expression of inflammatory factors, promote the differentiation of OPCs, and improve axonal hypomyelination. However, the mechanism of clemastine in improving axonal hypomyelination remains unknown. In vitro results showed that IL-1β administration delayed the development of oligodendrocytes, as evidenced by the decreased expression levels of MBP, CNPase, and Olig2 and as opposed to the increased expression levels of NG2. Clemastine, when administered in vitro, can revert this situation by promoting the maturation of primary OPCs in the IL-1β intervention. Thus, clemastine may trigger a signaling pathway to promote the expression of transcriptional factors, such as Olig2 which regulates the maturation of OPCs. Recently, the ERK signaling pathway has been identified to induce the maturation of OPCs [34]. The activation of ERK induces the expression levels of MBP and CNPase [18, 35]. Our previous research confirmed that IL-1β inhibited the maturation of OPCs by ERK phosphorylation inhibition [10]. The present research showed that treatment with clemastine promoted the phosphorylation of ERK insulted by IL-1β. Moreover, the expression levels of MBP and CNPase remarkably increased after clemastine treatment of the OPCs insulted by IL-1β for 7 days compared with those under IL-1β treatment. When OPCs treated with clemastine and IL-1β were added to SCH772984, the raise in MBP, CNPase, and Olig2 expression levels was reversed. Based on these results, the activation of ERK phosphorylation is suggested as one of the mechanisms by which clemastine promotes the maturation of OPCs. The release of IL-1β from the activated microglia leads to the damage of OPCs [10, 36]. Thus, preventing the excessive release of IL-1β is necessary. NLRP3 is the upstream signal factor controlling the release of IL-1β [37]; thus, inhibiting its release can reduce tissue damage [38]. MCC950 is a selective inhibitor of NLRP3 which reduces the release of IL-1β in vivo [39]. The present study revealed that the expression levels of NLRP3 and IL-1β increased after hypoxia. Furthermore, clemastine and MCC950 could reduce the excessive expression levels of NLRP3 and IL-1β, suggesting that clemastine and MCC950 featured an anti-inflammatory effect. Clemastine can inhibit the secretion of IL-1β by microglial cells [16], but the specific mechanism is unknown. According to reports, p38 is a signaling pathway regulating the IL-1β release [19]. Interestingly, the inhibition of p38 inhibits the secretion of IL-1β. SB203580 is a selective inhibitor of p38 signaling pathway [40]. In the present study, clemastine inhibited the NLRP3 and IL-1β expression levels, which were strengthened after the addition of SB203580 which inhibited p38. Co-culture of microglia with OPCs was established to verify the link between axonal hypomyelination and activation of microglial cells. The present research showed that the expression levels of MBP and CNPase remarkably increased after clemastine treatment of the OPCs insulted by OGD for 7 days compared with those under OGD treatment. When clemastine and OGD were added to SB203580, the raise in MBP and CNPase expression levels was strengthened, suggesting that clemastine alleviated hypomyelination by inhibiting the release of IL-1β via inhibition of the p38 signaling pathway. In summary, clemastine possibly improves axonal hypomyelination by a dual mechanism. The molecular mechanisms and signaling pathways by which clemastine improves axonal hypomyelination after ischemia–hypoxia are identified in the current study which may help in developing potential therapeutic strategies for axonal hypomyelination associated with HIBI.

## Conclusion

This study determined that following ischemia–hypoxia challenge in rats, the activated microglial cells (AMCs) in the corpus callosum produced excess amounts of IL-1β. The ensuing cellular interaction between AMCs and OPCs via IL-1β would impede the maturation of the latter cell type. This event was evidenced by the concurrent reduction in the formation of myelin sheaths. Clemastine improved axonal hypomyelination by inhibiting inflammation and promoting OPC differentiation. Further analysis revealed that clemastine promoted the maturation of OPCs through the activation of ERK phosphorylation in OPCs and inhibited the release of IL-1β by inhibiting the p38 pathway in AMCs. Further insights into the abovementioned process in which clemastine plays a pivotal role would be desirable for designing effective therapeutic strategies to ameliorate hypomyelination induced by HIBI.

## Supplementary information

**Additional file 1: Figure S1.** Position of immunofluorescence. **Figure S2.** Co-culture experiment with microglial cells and OPCs. **Figure S3.**The purity of microglial cells and OPCs.

## Data Availability

The datasets used and analyzed during the current study are available from the corresponding author on reasonable request.

## Abbreviations

AMCs: Activated microglia cells
BCCAO: Bilateral common carotid artery occlusion
FBS: Fetal bovine serum
HH1R: Histamine H1 receptor
HIBI: Hypoxic–ischemic brain injury
IL-1β: Interleukin-1beta
NLRP3: Nod-like receptor protein 3
OGD: Oxygen glucose deprivation
OPCDM: Oligodendrocyte precursor cell differentiation culture medium
OPCs: Oligodendrocyte progenitor cells
PBS: Phosphate-buffer saline
