# Supplementary material for: Clemastine improves hypomyelination in rats with hypoxic–ischemic brain injury by reducing microglia-derived IL-1β via P38 signaling pathway
Source: J Neuroinflammation. 2020 Feb 15;17:57. doi: 10.1186/s12974-019-1662-6 (PMC7023767; doi:10.1186/s12974-019-1662-6)

**Position of Immunofluorescence**


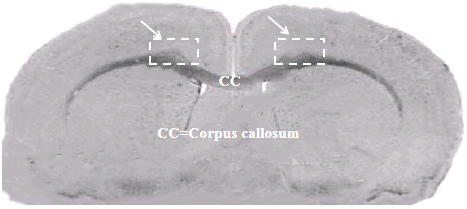


Brain illustration


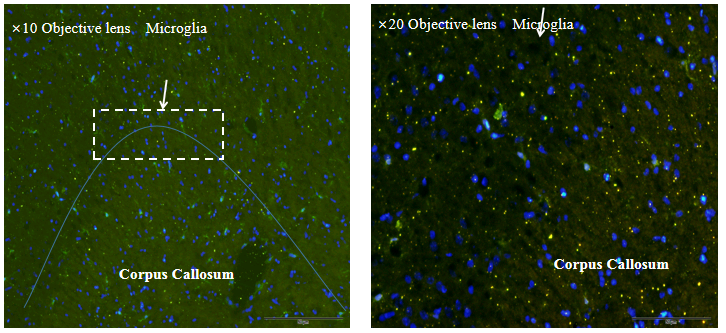


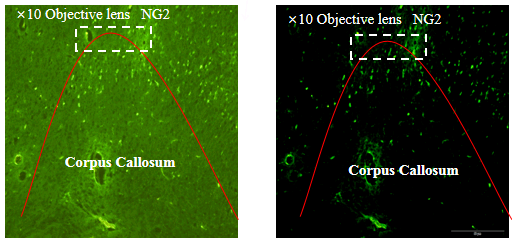


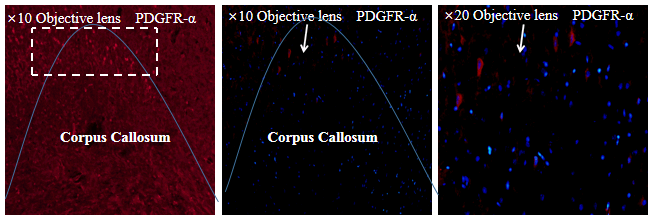


**Co-culture experiment with microglial cells and OPCs**


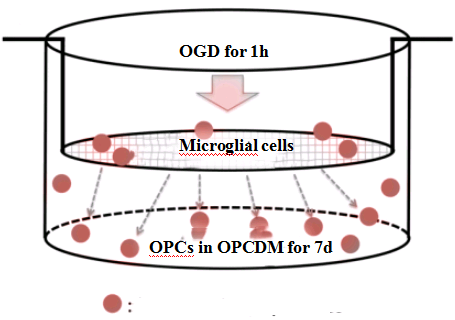


**IL-1β**

**The Purity of Microglial Cells**


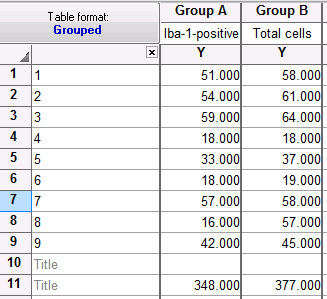

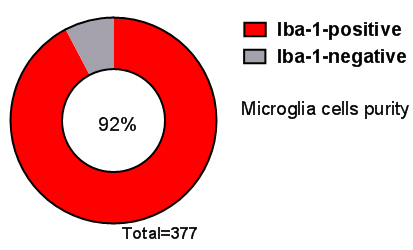

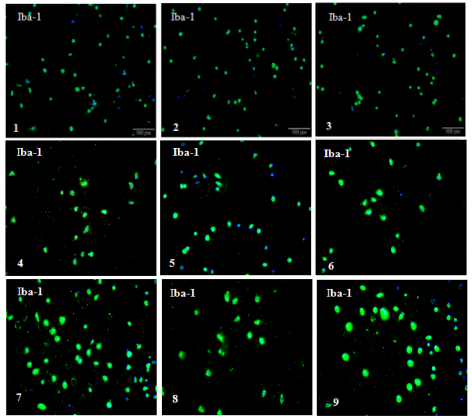


**The Purity of OPCs**


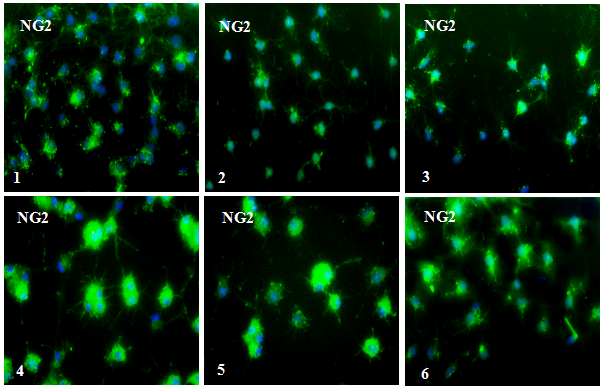


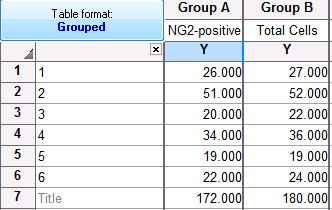

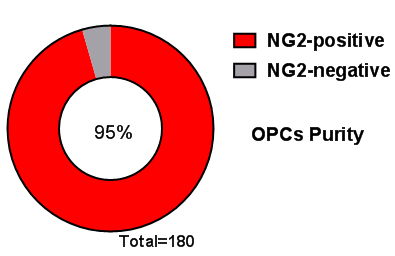

Supplement: Supplementary file 1 — Additional file 1: Figure S1. Position of immunofluorescence. Figure S2. Co-culture experiment with microglial cells and OPCs. Figure S3.The purity of microglial cells and OPCs. [file 12974_2019_1662_MOESM1_ESM.doc]
